# Neuropeptide y and neuropeptide s in major depressive disorder and post-traumatic stress disorder: Preclinical andclinical studies

**DOI:** 10.1192/j.eurpsy.2021.80

**Published:** 2021-08-13

**Authors:** A. Mathe

**Affiliations:** Clinical Neuroscience, Karolinska Institutet, Stockholm, Sweden

**Keywords:** Depression, Treatment, Neuropeptide y, intranasal

## Abstract

*MDD is the predominant cause of “Years of life lived with disability” and “Years of life lost because of premature death” as a consequence of the disorder per se, comorbidities (cardiovascular, diabetes) and the high suicide rate. The problem is increasing due to higher depression frequency with age and growing life-longevity. One third of patients do not respond adequately to conventional therapies and “more of the same” drugs will not solve the problem. Consequently, there exists a crucial need to develop treatments with different modes of action. *Neuropeptide Y (NPY) and neuropeptide S (NPS) have been mapped in brain of MDD and PTSD rodent models. NPY is reduced in genetic and environmental depression models and in PTSD and can be reversed by antidepressants. These findings are parallel to the decreased NPY in humans diagnosed with MDD and PTSD. *NPS found in locus caeruleus regulates anxiety and stress–related behaviors and intranasal administration is anxiolytic in rat. The intranasal effects in humans are being explored. *Based on known biology and our findings, we hypothesized that NPY could be a target for MDD and PTSD, the reasoning being analogous to insulin treatment in insulin deficient diabetes, and conducted - the first ever - double blind, placebo controlled trials of insufflated NPY in MDD and PTSD (Sayed et al 2018, Mathé et al 2020). In conclusion, intranasal NPY is opening a promising new avenue for efficient, fast acting treatment of MDD and PTSD. Support:The Swedish MRC #10414; Center Psychiatry Research-KI, The Torsten Söderbergs Stiftelse

**Disclosure:**

No significant relationships.

